# Straw-Based Activated Carbon: Optimization of the Preparation Procedure and Performance of Volatile Organic Compounds Adsorption

**DOI:** 10.3390/ma14123284

**Published:** 2021-06-14

**Authors:** Zhen Li, Yonghong Li, Jiang Zhu

**Affiliations:** 1 Key Lab for Green Chemical Technology of Ministry of Education, School of Chemical Engineering and Technology, Tianjin University, Tianjin 300072, China; zj_cnpe@163.com; 2 Department of Chemical Engineering, Tianjin University Renai College, Tianjin 301636, China; 3 National Engineering Research Center for Distillation Technology, Tianjin University, Tianjin 300072, China; 4 Collaborative Innovation Center of Chemical Science and Engineering, Tianjin University, Tianjin 300072, China

**Keywords:** straw, activated carbon, volatile organic compounds removal, optimization, response surface methodology

## Abstract

Straw is one of the largest agricultural biowastes and a potential alternative precursor of activated carbon. Activated carbon prepared from different types of straw have great differences in structure and adsorption performance. In order to explore the performance of different straw-based activated carbon in volatile organic compounds adsorption, five common straws were selected as potential source materials for the preparation of SAC. The straw-based activated carbons were prepared and characterized via a thermo-gravimetric analysis, scanning electron microscope and the Brunauer–Emmett–Teller method. Among the five straw-based activated carbons, millet straw-derived activated carbon exhibited superior properties in S_BET_, S_mic_ and adsorption capacities of both toluene and ethyl acetate. Furthermore, the preparation process of millet straw activated carbon was optimized via response surface methodology, using carbonization temperature, carbonization time and impregnation ratio as variables and toluene adsorption capacity, ethyl acetate adsorption capacity and activated carbon yield as responses. The optimal preparation conditions include a carbonization temperature of 572 °C, carbonization time of 1.56 h and impregnation ratio (ZnCl_2_/PM, *w*/*w*) of 1.60, which was verified experimentally, resulting in millet straw activated carbon with a toluene adsorption capacity of 321.9 mg/g and ethyl acetate adsorption capacity of 240.4 mg/g. Meanwhile, the adsorption isothermals and regeneration performance of millet straw activated carbon prepared under the optimized conditions were evaluated. The descriptive ability of the isothermals via the Redlich–Peterson equation suggests a heterogeneous surface on millet straw activated carbon. Recyclability testing has shown that millet straw activated carbon maintained a stable adsorption capacity throughout the second to fifth cycles. The results of this work indicate that millet straw activated carbon may be a potential volatile organic compound adsorbent for industrial application.

## 1. Introduction

Active carbons (ACs) have wide applications in environmental fields, such as groundwater [1], wastewater treatment [2] and volatile organic compounds (VOCs) control [3]. The demand for ACs is expected to grow at a CAGR of about 17.2% from 2019 to 2025 [4]. Various materials, including industrial residues, agricultural and forestry by-products and other low-cost resources, have been proposed for the preparation of ACs [5].

Straw from agricultural wastes is a promising resource for the production of ACs due to their low cost and the ability to avoid environmental pollution by incineration. Studies show that the straw-based activated carbons (SACs), prepared from rice straw [6,7,8], wheat straw [2,9,10], sesame straw [11], corn straw [12,13] and maize straw [10] had an adsorption effect on heavy metals and that the SACs, from corn straw [14,15] and wheat straw [14,16] can be used to remove organic dyes from water. SACs are also able to remove antibiotics [17,18,19].

VOCs are the major pollutants in the air caused by industrial development. The elimination of VOCs has gotten increasing public attention due to their adverse effects on both the environment and human health [20]. Adsorption of VOCs via ACs is attractive because of its low cost, simple processability and non-secondary waste disposal [21]. Furthermore, AC adsorption is non-deconstructive and provides the possibility of recover specific components in VOCs [22]. ACs produced from coconut shell [23], walnut shell [24] and peanut shell [25] have been investigated for removing toluene, benzene and other VOCs.

The application of SACs on VOC removal has also been reported. AC derived from maize straw was used to eliminate both NO and HgO from flue gas [26]. Biochar from rice straw was studied as a hydrogen [27] and CO_2_ [28] adsorbent. Wheat straw AC was employed to adsorb toluene and benzene [3]. Rice husk char was reported for toluene and phenol adsorption [29]. Considering the large variety of VOC components and the prospective market, studies of SACs on VOCs controlling remains limited and needs further efforts.

Furthermore, ACs derived from different sources exhibit varied adsorption characteristics in versatile applications. The content of lignin, cellulose and inorganic substances in the raw materials of activated carbon and the characteristics of surface functional groups make activated carbon with different void structures and surface characteristics, which determines the different adsorption characteristics of activated carbon products [30]. Meanwhile, pyrolysis temperature, carbonization temperature and impregnated chemicals can influence pore formation [22]. The properties of AC such as polarization, hydrophobicity, acidity and adsorption capacity could thus be tailored by appropriate carbonization and impregnation procedures [31].

The authors have reported the research on corncob-based AC for adsorption of toluene previously [32]. Subsequently, we compared several biowaste straws including corncob, peel of maize straw, millet straw, cotton straw and pepper straw, and have found that the AC made from millet straw showed the best adsorption performance for VOCs. In this paper, the preparation conditions of the millet straw-based AC were optimized by using response surface methodology (RSM) [33]. The adsorption isotherms and regeneration performance of this SAC prepared under optimized conditions were further investigated using toluene and ethyl acetate as model VOC components. The results of this work may help find a prospective cost-effective precursor for AC production toward VOC removal and environmental protection.

## 2. Materials and Methods

### 2.1. Materials

Five common straws, including the core of maize straw (CMS), peel of maize straw (PMS), millet straw (MS), cotton straw (CS) and pepper straw (PS) was collected from the Henan province, China. The raw straws were fully washed with distilled water, dried at 110 °C for 48 h, crushed and sieved to the size range of 0.250–0.600 mm before use. Toluene (>99.5 wt%), ethyl acetate (>99.5 wt%) and ZnCl_2_ (>98.0 wt%) were supplied by Fengchuan Chemical Reagent Co., Ltd., Tianjin, China. Nitrogen and air were purchased from Liufang Gases Chemical Reagent Co., Ltd., Tianjin, China.

### 2.2. Preparation of SACs

SACs were prepared using the modified method based on the article [32]. In each experiment, 0.5 g of straw was thoroughly mixed with and soaked in 4.0 mL of solution containing 0.5 g of ZnCl_2_ at an ambient temperature for 8 h. Zinc chloride is a classic chemical activator, which has the role of catalytic dehydroxyl and dehydration, so that the hydrogen and oxygen in the raw material is released in the form of water vapor, forming a porous structure [34,35,36]. The mixture was then dried at 110 °C for 12 h to get impregnated samples. Sequentially, the impregnated samples were heated to the preset temperature at a rate of 20 °C∙min^−1^ and maintained at that temperature for 1 h in a 316 stainless steel tubular reactor under N_2_ atmosphere. After being cooled, the as-prepared AC samples were washed with 1.0 mol∙L^−1^ of hydrochloric acid solution at 50 °C for 30 min to remove metal ions and ash content, and with hot deionized water repeatedly until the pH of the filtrate reached 7. The washed AC samples were then dried at 80 °C under vacuum for 12 h, followed by being ground/sieved to the size range of 0.180–0.425 mm. The final AC samples were labeled as XSAC, with X standing for the source straws.

### 2.3. SACs Characterization

The pyrolysis behaviors of the straws were investigated using a thermogravimetric data analysis (TG209F3, NETZSCH, GER, Selbu, Norway). The morphology of the as-prepared SACs was observed using a field emission scanning electron microscope (SEM) (Nanosem 430, FEI, Hillsboro, OR, USA).

The textural characteristics of SAC samples were determined by physical N_2_ adsorption–desorption at 77K using an auto-adsorption system (Autosorb-iQ2-MP, Quantachrome, Boynton Beach, FL, USA). The specific surface area (S_BET_) was calculated through N_2_ adsorption isotherm using the Brunauer–Emmett–Teller (BET) equation. The external surface area (S_ext_) and micropore volume (V_mic_) were determined using the t-plot method [37,38,39]. The microporous specific surface area (S_mic_) was calculated through the difference between S_BET_ and S_ext_. The total pore volume (V_t_) was defined as the liquid N_2_ volume adsorbed at the relative pressure of 0.99. The mesopore volume (V_mes_) was calculated through the difference between V_t_ and V_mic_. Pore size distribution was calculated using the DFT method.

### 2.4. Adsorption and Regeneration Evaluation

**Figure 1:**
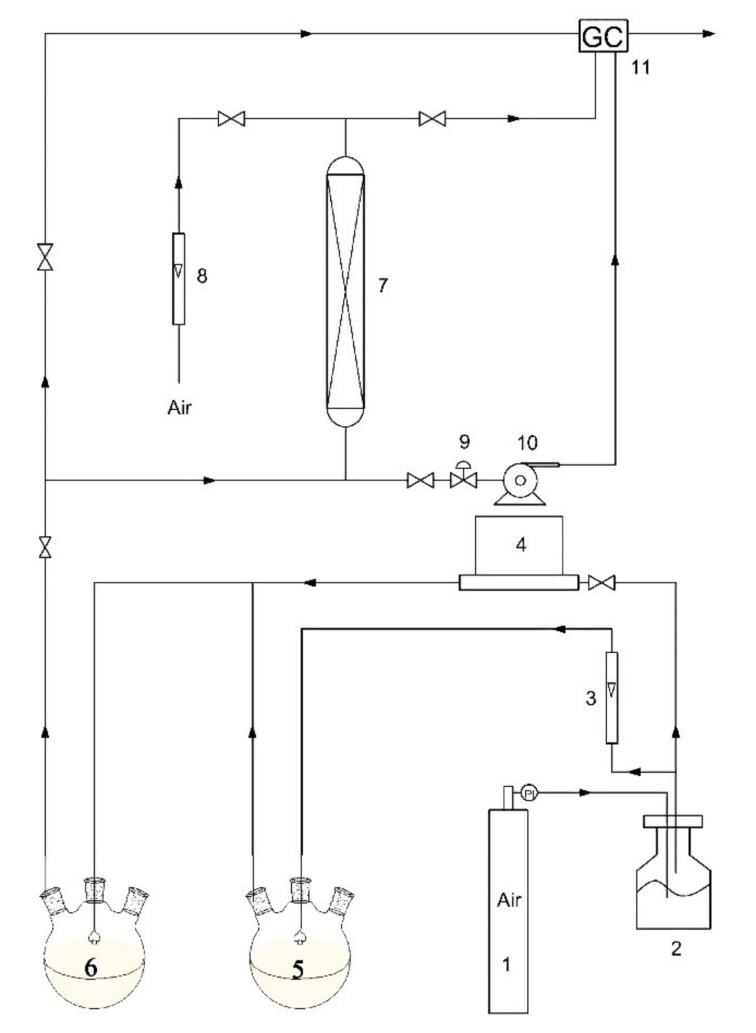
Experimental device diagram (1: Air cylinder; 2: Silica gel dryer; 3: Rotameter; 4: Mass flowmeter; 5: VOC generator; 6: Buffer bottle; 7: Adsorption column; 8: Rotameter; 9: Vacuum degree regulating valve; 10: Vacuum pump; 11: Gas chromatography).

The adsorption and desorption experiments of SACs were carried out on a device consisting of three parts: VOCs gas generation system, thermostatic adsorption system and concentration determination system. The flow diagram of the experimental device is shown in Figure 1. Toluene and ethyl acetate, as typical VOC components, were selected as adsorbates to evaluate the adsorption capacities of the as-prepared SACs.

In the VOCs gas generation system, the carrier gas/air was divided into two streams via a 3-way valve. One stream was led into toluene/ethyl acetate to generate VOC vapor, and the other stream was used to adjust the concentration of VOC vapor in the combined streams by tuning the flow ratio of the two streams. The total flow rate of the two streams in all the experiments was fixed at 500 mL/min.

The thermostatic adsorption system contains a U-shape glass adsorption column, which can load 0.3 g of AC. The adsorption column was placed in a water bath to control the temperature.

The concentration determination system contains a gas chromatography (GC, FULI 9790, Wenling, China) equipped with a flame ionization detector (FID). The VOC concentration was determined by the standard curve method using GC before and after the adsorption. The standard curves of toluene and ethyl acetate were measured by correlating the relationship between the concentration and the corresponding peak area.

After the VOCs gas concentration was kept constant, it was introduced into the adsorption column to start the experiments. The concentration of outlet VOC gas was measured at intervals until it was equal or close to the inlet gas concentration, indicating the SACs were saturated with VOCs. Then the adsorption capacity of the SACs was calculated as follows:

(1) $$q_{m}=\frac{F}{m}\left(C_{o}t-\overset{t}{\underset{0}{\int }}C_{i}dt\right)$$

where *q_m_* is the adsorption capacity (mg∙g^−1^); *F* is the total flow rate of the VOC gas (m^3^∙min^−1^); *C*_0_ and *C_i_* are the inlet and outlet gas concentrations (mg∙m^−3^); *m* is the mass of the adsorbents (g); *t* is the adsorption equilibrium time (min).

The regeneration of the SACs was performed under vacuum. During regeneration, the VOC gas was turned off, and hot air was introduced into the adsorption system as the purge gas. The concentration of the outlet gas was monitored by GC. The desorption ratio was calculated as follows:

(2) $$Desorption\;ratio=\frac{m_{d}}{m_{a}}$$

where *m_a_* is the total weight of the adsorbed VOCs and m_d_ is the weight of the desorbed VOCs during regeneration.

### 2.5. Optimization of the Preparation Conditions for MSAC via RSM

The preparation parameters of MSAC were optimized to maximize the responses, i.e., toluene adsorption capacity (*Y_1_*_)_, ethyl acetate adsorption capacity (*Y_2_*) and AC yield (*Y_3_*) by RSM with three independent factors including carbonization temperature (*A*), carbonization time (*B*) and impregnation ratio (*C*). The experiments were designed with five levels including +1.68 (extremely strong), +1 (strong), 0 (middle), −1 (weak) and −1 (extremely weak). The five levels of factor *A* were 350, 450, 550, 650 and 750 °C. The factor *B* varied as 0.5, 1.0, 1.5, 2.0 and 2.5 h. The factor *C* was adjusted as 0.5, 1.0, 1.5, 2.0 and 2.5.

## 3. Results

### 3.1. Pyrolysis Behavior of Different Straws

**Figure 2:**
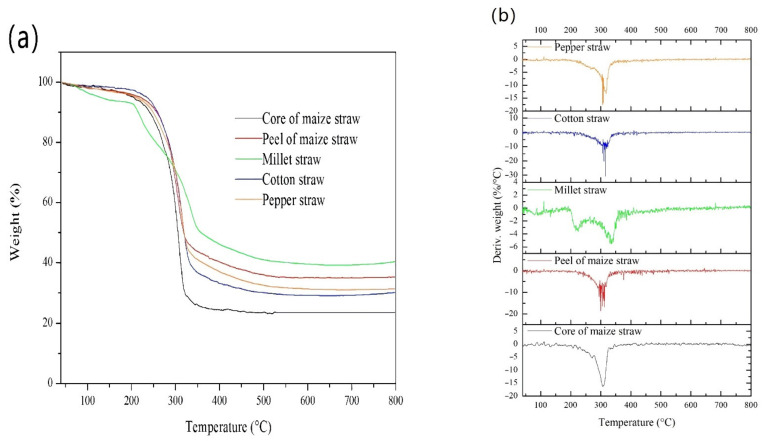
The pyrolysis behaviors of the five straws: (**a**) TG curves; (**b**) DTG curves.

The preparation of biomass-based AC usually contains pyrolysis and activation. The pyrolysis behaviors of the five straws are given in Figure 2 using TG data analysis. All the straws used in this work, except MS, exhibited similar TG and DTG curves. The pyrolysis of these four straws can be divided into three stages according to the weight loss (Figure 2a). The first stage occurred at 40–200 °C, during which the weight loss was less than 5%. The small weight loss can be attributed to moisture removal. Most of the weight loss happened in the second stage, which occurred at 200–350 °C. The weight losses were 50.2% for PMS, 51.7% for PS, 53.5% for CS and 68.9% for CMS. The corresponding endothermal peaks of these four straws in this stage were all at about 300 °C, which can be attributed to the pyrolysis of cellulose, hemicellulose and lignin [40,41]. A temperature over 350 °C corresponded to the third stage with small weight loss, which may be attributed to the slow gasification of small quantities of intermediate products of pyrolysis.

As for MS, the pyrolysis behavior was apparently different. The main second pyrolysis stage of MS was separated into two steps. One step occurred at 200–300 °C with a weight loss of 22%, corresponding to the decomposition of hemicellulose. The other step happened at 300–350 °C with a weight loss of 20%, which could be attributed to the decomposition of cellulose. It suggests that MS contains more hemicellulose components than the other four straws used in this work.

Figure 2 shows that MS has the lowest starting temperature and broadest temperature window of carbonization among the five straws. The carbonization of the five straws all complete at lower than 600 °C. Figure 2a also gives the AC yield according to the residue weight fraction at 800 °C. It suggests that the yield order of the five straws is MS > PMS > PS > CS > CMS, among which MS has the highest yield of about 40.3%.

### 3.2. Characterization of SACs

**Figure 3:**
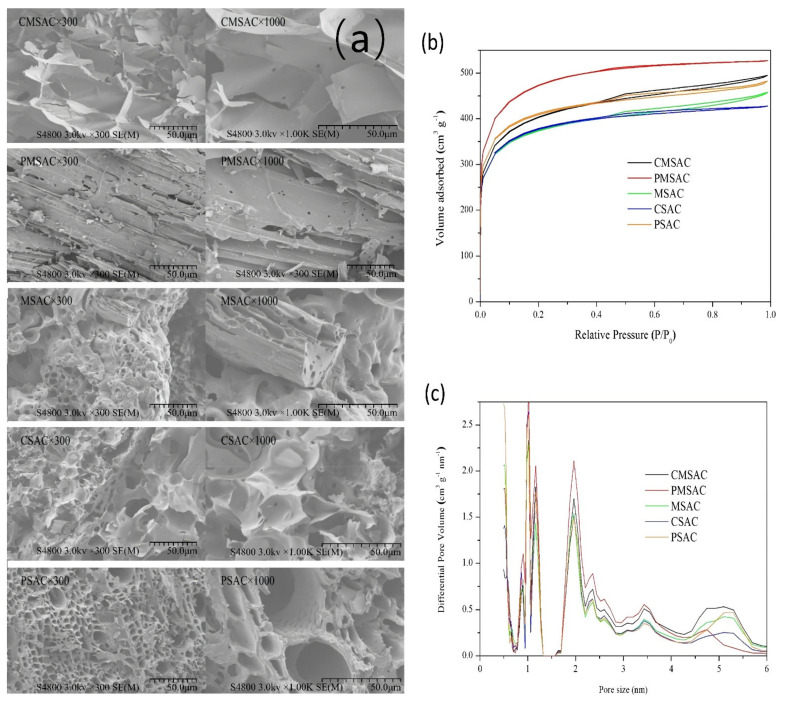
Characterization of different SACs: (**a**) SEM images, (**b**) N2 adsorption–desorption isotherms and (**c**) pore size distributions.

The SEM images (Figure 3a) of the five as-prepared SACs were characterized to observe the pores and surface morphology. The surfaces of CMSAC and PMSAC were relatively smooth, while that of CSAC and PSAC were rough, owing to different reactivity and the distribution of different components. The N_2_ adsorption–desorption isotherms (Figure 3b) were determined to evaluate the porosity of the five SACs. The PMSAC exhibits typical type I isotherm characteristics [42], which correspond to the Langemue equation. When approaching the saturated vapor pressure, the isotherm rises rapidly due to the presence of gaps between the particles, which are similar to microporous adsorption. The type I adsorption isotherm indicates that PMSAC is a microporous material. The isotherms of the other four samples are similar in that the adsorption capacity rises rapidly at low relative pressure, and the curve is convex. The reason is that there is a strong interaction between the adsorbate and the surface. The hysteresis loop in the middle segment corresponds to the capillary condensation of the porous adsorbent, which belongs to type IV adsorption isotherms, indicating the existence of mesopores. [43].

The pore size distributions of all five SAC samples (Figure 3c) were then measured. It shows that all the five SACs have multimodal pore size distributions within the range of mesopore and micropore (<6 nm), which provide a high specific surface area. Among the five SACs, PMSAC has the most mesopores and micropores (<4.5 nm) and the least big mesopores (~4.5–6 nm).

The detailed surface area and porosity volume of the SACs are presented in Table 1. The S_BET_ and S_mic_ both follow the order of PMSAC > MSAC > CMSAC > CSAC > PSAC. The orders of V_t_ and V_mic_ are PMSAC > CMSAC > MSAC > PSAC > CSAC, which are different from that of S_BET_ and S_mic_. Among the five SACs, the PMSAC exhibits the largest S_BET_, V_t_, S_mic_ and V_mic_. In general, the surface area and pore volume are dominated by micropores, which are considered the main adsorption sites of VOC components. Thus, PMSAC may have the best adsorption properties among the five SACs in this work.

### 3.3. Adsorption Capacity Study

The adsorption capacities of each SAC for toluene and ethyl acetate in Table 1 were tested at 25 °C to explore their application potential in VOC adsorption. The order of the adsorption capacity for toluene is PMSAC > MSAC > PSAC > CMSAC > CSAC, which is almost the same as the order of S_BET_ and S_mic_. In the adsorption of ethyl acetate, the adsorption capacity order is PMSAC > MSAC > PSAC > CSAC > CMSAC, which roughly follows the order of S_BET_ and S_mic_. Notably, CMSAC and CSAC have very similar S_BET_, S_mic_ and adsorption capacities for toluene and ethyl acetate. All these results suggest that the adsorption might occur mainly on the surface of micropores. The only exception is MSAC, which has relatively fewer micropores but a higher adsorption capacity. It implies that MSAC may provide stronger interactive adsorption sites for toluene/ethyl acetate than other SACs. However, the difference between the orders of adsorption capacities and microporosity may be attributed to the fact that the adsorption capacity was based on VOC adsorption while microporosity analysis was based on N_2_ adsorption. Therefore, it suggests that the interaction between adsorbent and adsorbate, as well as the microporosity, is also important to the adsorption capacity.

In addition, the adsorption capacity for toluene was found to be higher than that for ethyl acetate on each SAC. The difference is much bigger than the difference between the molecular weight/size of toluene and ethyl acetate. The reason why more toluene molecules, compared to ethyl acetate, can be adsorbed on the SACs may be the strong π-π interaction between toluene and the carbon surface.

### 3.4. RSM Optimization of MSAC Preparation

According to the adsorption capacity study (Section 3.3), PMSAC and MSAC show higher performance on both toluene and ethyl acetate adsorption than the other three SACs in this work, but the AC yield of PMS is lower than MS. The comprehensive performance of MSAC is the best among the five SACs if considering both yield and adsorption capacity. We thus chose MSAC to further optimize the preparation conditions and evaluate its performance on VOC adsorption.

#### 3.4.1. Experimental Results of RSM

RSM is a powerful statistical tool that can understand multiple parameters in a complex process. The parameters of the process could be optimized by fitting the factors and responses via multiple quadratic regression equations [33].

The experimental results of the responses (*Y*_1_, *Y*_2_ and *Y*_3_) to three independent preparation condition factors (*A*, *B* and *C*) are listed in Table 2. The relationships between the responses and the factors were correlated via regress analysis using multiple quadratic equations. The final regression equations of *Y*_1_, *Y*_2_ and *Y*_3_ are given in Equations (3)–(5). A positive coefficient indicates a synergistic effect, while a negative coefficient suggests an antagonistic effect.

(3) $$Y_{1}=367.72+39.71A-4.21B+8.71C+7.18AB+1.73AC-10.2BC-39.94A^{2}-4.78B^{2}-13.12C^{2}$$

(4) $$Y_{2}=247.40+24.37A+2.49B+10.80C-0.64AB+5.16AC-4.91BC-33.48A^{2}-8.31B^{2}-23.03C^{2}$$

(5) $$Y_{3}=36.48-2.50A-0.28B-0.23C+0.25AB-0.45AC+0.88A^{2}-0.11B^{2}+0.7C^{2}$$

The analysis of variance (ANOVA) was taken to determine the reliability of the fittings. According to the ANOVA data (Please see the Supplementary Materials Tables S1–S3), the *p* (lack of fit) values of the three models are all less than 0.0001, indicating that the model fitting is significant [44]. Meanwhile, the F values of (lack of fit) are all greater than 0.05 (0.1129, 0.16 and 1.27, respectively), indicating that the lack of fit is not significant (the models are all statistically significant). The large R^2^ of the equations (0.9358, 0.8614 and 0.9346) indicates a good correlation of the experimental data using Equations (3)–(5). The small C_V_ values (5.55%, 11.2% and 2.31%) suggest that the experimental operations are reliable.

The significance testing results of each regression model show that:

(1) For toluene adsorption capacity, *Y*_1_, the significant terms are the linear terms of *A* and *C*, and quadratic terms of *A*^2^ and *C*^2^. The effects of *A* and *C* are positive, while that of *A*^2^ and *C*^2^ are negative. *A* and *A*^2^ have the greatest impact on *Y*_1_. *B* has little influence on *Y*_1_. This means that the carbonization temperature, *A*, is the most important factor and is optimal for toluene adsorption capacity, *Y*_1_.
(2) For the adsorption capacity of ethyl acetate, *Y*_2_, the most significant terms are the linear terms of *A* and *C* and quadratic terms of *A*^2^ and *C*^2^, which are similar to *Y*_1_. Notably, *C*^2^ has a much greater effect on *Y*_2_ than on *Y*_1_, which means the impregnation ratio, *C*, needs more attention for ethyl acetate adsorption during the optimization. All the interaction terms have little influence on *Y*_2._
(3) For the yield of SAC, *Y*_3_, the carbonization temperature, *A*, is the only significant term that has a negative impact on *Y*_3._ This means the yield of SAC decreases with increasing carbonization temperature.

#### 3.4.2. RSM Analysis

**Figure 4:**
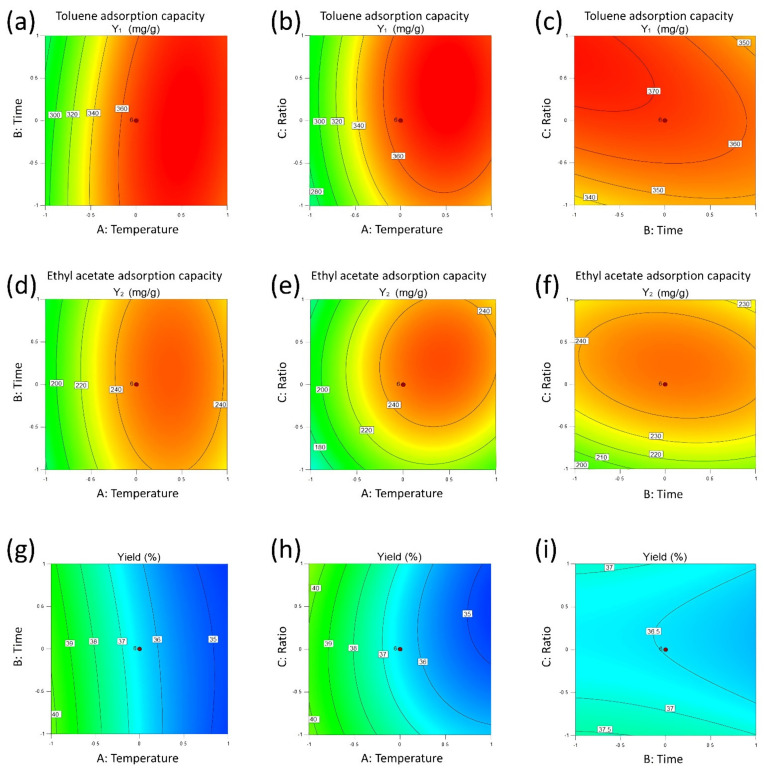
Contour maps of surface response plots for toluene adsorption capacity (**a**–**c**), ethyl acetate adsorption capacity (**d**–**f**) and carbon yield (**g**–**i**).

Figure 4a–c shows the contour diagrams of surface response plots for toluene adsorption capacity according to Equation (3). Figure 4a shows the effects of carbonization temperature and carbonization time on the adsorption capacity of toluene with a central impregnation ratio. It is obvious that the adsorption capacity for toluene is sensitive to the carbonization temperature, especially at low-temperature ranges where the adsorption capacity for toluene increased rapidly with increasing carbonization temperature. When the carbonization temperature was higher than 0.5, the adsorption capacity for toluene slightly declined but still remained at a high level. Carbonization time, however, is not a sensitive factor in the tested time range, implying a fast reaction and pore amplification process. Figure 4b,c indicates that the impregnation ratio had the largest positive effect on the adsorption capacity for toluene at a high level (about 0.5). The elliptical isohypse lines shown in Figure 4c indicates that the carbonization temperature and carbonization time have significant interaction.

Figure 4d–f shows the contour diagrams of surface response plots for ethyl acetate adsorption capacity according to Equation (4). The effects of the interaction between carbonization temperature and carbonization time and the impregnation ratio on the adsorption capacity for ethyl acetate was similar to that on the adsorption capacity for toluene. Raising either carbonization temperature or impregnation ratio can first enhance and then decrease the ethyl acetate adsorption capacity. Carbonization time and impregnation ratio have a significant interaction according to Figure 4d,e. The ethyl acetate adsorption capacity was not sensitive to the carbonization time (Figure 4a,c). The difference between those effects on the adsorption capacity of toluene and ethyl acetate may rely on the different demands on pore size distribution for the two adsorbates.

Figure 4g–i shows the contour diagrams of surface response plots for SAC yield according to Equation (5). Figure 4g,h show that the yield of MSAC decreased gradually with increasing carbonization temperature, which could be attributed to the pyrolysis of biomass at high temperatures. Figure 4g–i also shows that the variation range of AC yield was very small, indicating that carbonization time and impregnation ratio are not sensitive to the yield of MSAC.

The 3D surface of surface response plots for toluene adsorption capacity, ethyl acetate adsorption capacity, and carbon yield shows in Figure S1 which provided in Supplementary Materials.

#### 3.4.3. Parameter Optimization and Verification

The adsorption capacities of PMSAC for toluene and ethyl acetate were optimized simultaneously by using Design-Expert software. The optimized conditions were carbonization temperature of 565 °C, carbonization time of 1.26 h and impregnation ratio of 1.55, under which the predicted optimal responses were the toluene adsorption capacity of 368.5 mg/g and the ethyl acetate adsorption capacity of 241.9 mg/g.

To verify the optimization, MSAC was prepared under the optimized conditions to determine the adsorption capacity for toluene and ethyl acetate. The experimental adsorption capacities for toluene and ethyl acetate were 360.4 and 248.8 mg/g, respectively, which were in good agreement with the predicted optimal with a small relative error of 2.24% and 2.77%. The small errors confirm the validation of the models proposed using RSM.

### 3.5. Adsorption Isotherms of Toluene and Ethyl Acetate

#### 3.5.1. Adsorption Isotherms of Toluene and Ethyl Acetate on MSAC

**Figure 5:**
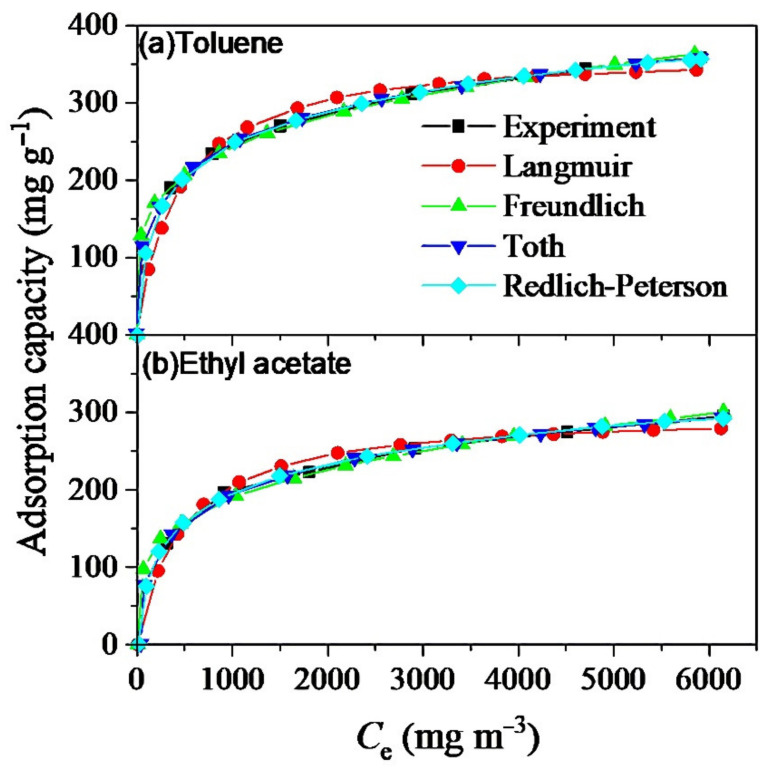
Adsorption isotherms of toluene (**a**) and ethyl acetate (**b**) on MSAC.

The adsorption isotherm is of great significance for revealing the interaction between the adsorbent and the adsorbate and expanding the application of adsorbents in the industry. Isothermal adsorption data of toluene and ethyl acetate on MSAC was determined under the conditions of adsorption temperature of 25 °C, VOC concentration in the range of 500–6000 mg/m^3^ and feed gas flow rate of 500 mL/min. The equilibrium adsorption capacity (q_e_) was measured at the equilibrium concentration (C_e_) between 500 and 6000 mg/m^3^ of the adsorbate. The adsorption data were fitted by various models, including the Langmuir equation, Freundlich equation, Sips equation, Toth equation and Redlich–Peterson equation. The adsorption isotherms of toluene and ethyl acetate obtained via experiment and fitting are shown in Figure 5.

The brief introduction of the Langmuir equation, Freundlich equation, Sips equation, Toth equation and Redlich–Peterson equation is listed in the Supporting Information. The relevant parameters are listed in Table 3.

The correlation coefficients, R^2^, of all the adsorption isotherms are greater than 0.98. The Freundlich adsorption isotherm model has a larger R^2^ than that of the Langmuir model for both toluene and ethyl acetate, indicating that MSAC may have a heterogeneous surface rather than a homogeneous surface for the adsorption on MSAC [45]. The Sips, Toth and Redlich–Peterson models fit the data better than the Freundlich and Langmuir equations, among which the Redlich–Peterson model has the highest fitting R^2^. This suggests that the adsorption of toluene and ethyl acetate on MSAC is between Langmuir adsorption and Freundlich adsorption.

#### 3.5.2. Regeneration of MSAC

Regeneration of used adsorbents is important to reduce both the adsorbent cost and disposal of waste materials cost. The regeneration experiments via desorption were carried out on the VOC-saturated MSAC with different desorption pressures and flow rates of purge. The desorption ratios at fixed a desorption temperature (65 °C) within 40 min are given in Table 4.

The desorption ratios of toluene and ethyl acetate on MSAC both increased with decreasing desorption pressure and increasing purge air flow under the fixed desorption temperature. This is reasonable because a low desorption pressure can promote the desorption thermodynamically, and a high purge air flow rate can accelerate the desorption kinetics of VOCs from the adsorbent.

For toluene, a 12.1% increase in desorption ratio was found by decreasing the desorption pressure from 34 to 11 kPa at the purge air flow rate of 0.2 L/min. Whereas an 18.2% increase in desorption ratio was obtained when increasing purge air flow rate to 1 L/min at 34 kPa. The same trend was observed for ethyl acetate that the corresponding increase of desorption ratio at the same condition changes were 7.0% and 15.7%, respectively. It concludes that, for both toluene and ethyl acetate, the desorption ratio on PMSAC is more sensitive to purge air flow rate than to desorption pressure. However, the changes of desorption ratios within the variable ranges of this work are not big, suggesting that, in industrial production, MSAC desorption should be carried out under proper vacuum and purge gas velocity to reduce the cost.

The desorption ratios of toluene and ethyl acetate were 66–78% and 70–82%, respectively. Under the same conditions, the desorption ratio of ethyl acetate is always higher than that of toluene. The reason may be due to the following respects. On the one hand, the interaction between toluene and MSAC is stronger than that between ethyl acetate and MSAC, which is consistent with the reason for the adsorption capacities order. On the other hand, ethyl acetate has a smaller molecular diameter than toluene and thus has a smaller desorption hindrance [46].

**Figure 6:**
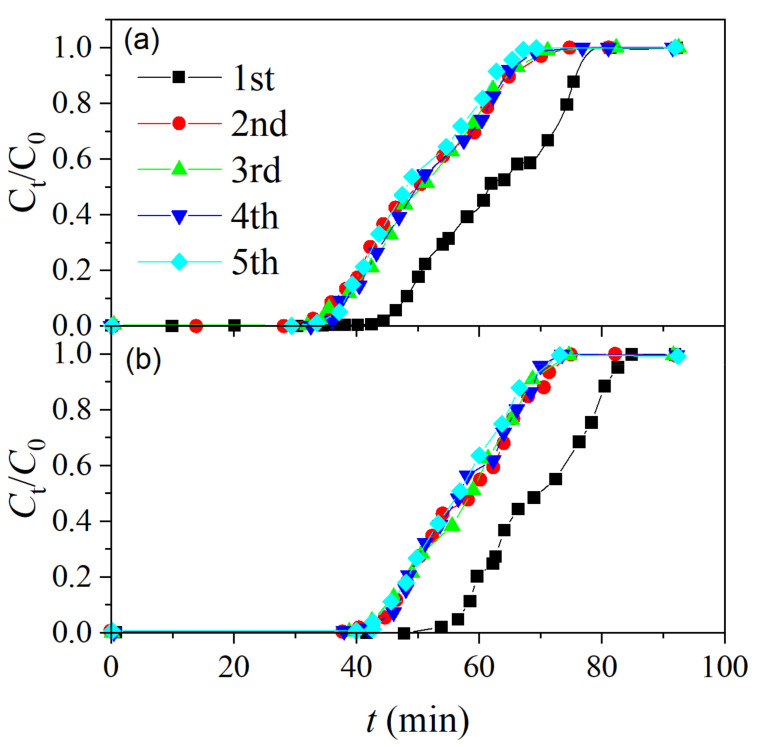
Breakthrough curves in five cycles of adsorption/desorption: (**a**) toluene; (**b**) ethyl acetate.

To investigate the regeneration performance and recyclability of MSAC, five adsorption/desorption cycles were conducted at 65 °C and 11 kPa with a purge air flow rate of 0.6 L/min. The experimental breakthrough curves of toluene and ethyl acetate are shown in Figure 6.

Toluene and ethyl acetate show similar breakthrough curve features. Both the onset time and the saturation time occurred about 15 min earlier after the first cycle, implying that VOCs cannot be totally removed during the regeneration. Notably, during the second–fifth cycles, the adsorption and desorption processes changed very little, and MSAC exhibited a constant adsorption capacity for toluene and ethyl acetate. It demonstrates that MSAC has a long, effective adsorbent life and may be a potential alternative VOC adsorbent for industrial production.

## 4. Conclusions

Five straw-based activated carbons from common straws were investigated for adsorption of volatile organic compounds. Millet straw activated carbon (MSAC) exhibited superior properties in S_BET_, S_mic_ and adsorption capacity. The preparation conditions of MSAC were further optimized by using response surface methodology (RSM). Multiple quadratic equations gave good correlations of the relationships between the condition variables and the corresponding responses, i.e., adsorption capacity, ethyl acetate adsorption capacity and AC yield.

The optimal conditions were found to be a carbonization temperature of 572 °C, carbonization time of 1.56 h and impregnation ratio of 1.60, with a predicted toluene adsorption capacity of 321.9 mg/g and the ethyl acetate adsorption capacity of 240.4 mg/g.

MSAC prepared under the optimized conditions exhibited experimental adsorption capacity for toluene of 316.1 mg/g and for ethyl acetate of 251.7 mg/g. The results are very close to the optimization via response surface methodology, with errors of 1.82% and 4.49%, respectively.

The analysis of the isotherms of toluene and ethyl acetate on millet straw activated carbon shows that the heterogeneous surface of millet straw activated carbon is available for adsorption. The regeneration of millet straw activated carbon via desorption based on thermal methods at 65 °C shows that the desorption ratios of toluene and ethyl acetate were 66–78% and 70–82%. Desorption pressure has more of an effect than purge air flow rate on the desorption ratio. However, the effects of those two factors were not significant within the condition range in this work.

The recyclability test shows that millet straw activated carbon encountered a decline in saturation time after the first cycle of adsorption–desorption experiments but maintained a stable adsorption capacity in the second–fifth cycles. The result indicates that millet straw activated carbon could be a potential volatile organic compound adsorbent for industrial application.

## Figures and Tables

**Table 1:** The textural characteristics and adsorption capacities of SACs.

Sample	Specific Surface Area (m^2^ g^−1^)			Pore Volume (cm^3^ g^−1^)			Saturation Adsorption Capacity (mg/g)	
	S_BET_	S_mic_	S_ext_	V_t_	V_mic_	V_mes_	Toluene	Ethyl Acetate
CMSAC	1474	1336	137.5	0.765	0.551	0.215	369.5 ± 7.2	217.4 ± 10.2
PMSAC	1733	1632	100.4	0.815	0.690	0.125	419.5 ± 3.8	297.5 ± 3.9
MSAC	1515	1413	102.0	0.731	0.581	0.150	376.1 ± 11.7	260.7 ± 8.4
CSAC	1390	1302	88.04	0.622	0.541	0.122	364.6 ± 9.0	250.0 ± 13.5
PSAC	1378	1265	112.5	0.708	0.518	0.190	374.2 ± 13.8	252.8 ± 6.7

**Table 2:** Design of variables and experiment data of RSM.

No.	Variables			Experimental Value		
	*A*	*B*	*C*	*Y_1_* (mg/g)	*Y_2_* (mg/g)	*Y_3_* (%)
1	0	0	0	372.9	257.8	35.1
2	−1	1	1	284.7	189.4	39.4
3	−1	1	−1	269.1	147.9	39.4
4	0	0	0	376.3	257.2	36.8
5	0	0	1.68	361.5	201.8	38.9
6	−1	−1	1	301.1	162.5	40.4
7	0	0	0	373.7	262.9	37.3
8	0	0	0	369.6	255.4	36
9	0	−1.68	0	365.9	210.6	37
10	1	1	−1	339.2	198.3	36.5
11	0	1.68	0	357.7	240.9	35.9
12	−1.68	0	0	203.5	120.8	44.3
13	−1	−1	−1	247.1	148.2	40.4
14	1.68	0	0	321.2	188.3	34.2
15	1	−1	1	349.4	236.1	34.7
16	1	−1	−1	342.3	186.8	36.5
17	0	0	−1.68	314.9	166.4	38.6
18	0	0	0	371.6	270.1	36.5
19	0	0	0	368.9	263.7	37.1
20	1	1	1	359.3	213.6	34.7

Note: *A* refers to carbonization temperature, *B* refers to carbonization time and *C* refers to impregnation ratio; *Y*_1_ refers to toluene adsorption capacity, *Y*_2_ refers to ethyl acetate adsorption capacity and *Y*_3_ refers to activated carbon yield.

**Table 3:** Values of adsorption isotherm constants.

Adsorption Isotherm	Constants	Materials	
		Toluene	Ethyl Acetate
Langmuir	$q_{m}$	373.7	304.0
	$K_{A}$	0.0022	0.0021
	R^2^	0.9899	0.9890
Freundlich	$k_{f}$	53.18	39.83
	n	0.2245	0.2295
	R^2^	0.9989	0.9971
Sips	$a_{m}$	647.8	511.2
	$K_{S}$	0.0496	0.0352
	n	0.3962	0.4181
	R^2^	0.9998	0.9982
Toth	f	872.8	660.3
	g	1.512	1.898
	d	0.2241	0.2458
	R^2^	0.9997	0.9983
Redlich–Peterson	$K_{R}$	2.8835	1.913
	$\alpha_{R}$	0.0342	0.028
	β	0.8293	0.8302
	R^2^	0.9999	0.9985

**Table 4:** Desorption ratio (%) of toluene and ethyl acetate at different flow rates of purge gas and desorption.

Flow Rate of Purge Gas (L/min)	Desorption Pressure (kPa)					
	11		21		34	
	Toluene	Ethyl Acetate	Toluene	Ethyl Acetate	Toluene	Ethyl Acetate
0.2	74 ± 2	75 ± 5	71 ± 3	73 ± 2	66 ± 2	70 ± 1
0.6	77 ± 3	81 ± 3	76 ± 4	80 ± 2	76 ± 2	77 ± 1
1.0	78 ± 1	82 ± 2	78 ± 2	82 ± 1	78 ± 1	81 ± 3

## Data Availability

Data is contained within the article or Supplementary Materials.

## Supplementary Materials

The following are available online at https://www.mdpi.com/article/10.3390/ma14123284/s1, Table S1 Analysis of variance (ANOVA) of toluene adsorption capacity(Y1); Table S2. Analysis of variance (ANOVA) of ethyl acetate adsorption capacity(Y2); Table S3. Analysis of variance (ANOVA) of yield(Y3); Figure S1: 3D surface of surface response plots for toluene adsorption capacity (a–c), ethyl acetate adsorption capacity (d–f), and carbon yield (g–i).
