# Supplementary material for: Straw-Based Activated Carbon: Optimization of the Preparation Procedure and Performance of Volatile Organic Compounds Adsorption
Source: Materials (Basel). 2021 Jun 14;14(12):3284. doi: 10.3390/ma14123284 (PMC8232107; doi:10.3390/ma14123284)
Supplement: Supplementary file 1 [file materials-14-03284-s001.zip › materials-1250410-supplementary.pdf]

materials

MDPI

Support Information

Straw-Based Activated Carbon: Optimization of the Preparation Procedure and Performance of Volatile Organic Compounds Adsorption

Zhen Li [1,2,3,4,*], Yonghong Li [1,3,4,*] and Jiang Zhu [1,3]

[1] Key Lab for Green Chemical Technology of Ministry of Education, School of Chemical Engineering and Technology, Tianjin University, Tianjin 300072, China; zj_cnpe@163.com
[2] Department of Chemical Engineering, Tianjin University Renai College, Tianjin 301636, China
[3] National Engineering Research Center for Distillation Technology, Tianjin University, Tianjin 300072, China
[4] Collaborative Innovation Center of Chemical Science and Engineering, Tianjin University, Tianjin 300072, China
* Correspondence: jane_lee@tju.edu.cn (Z.L.); yhli@tju.edu.cn (Y.L.); Tel.: +86-150-2230-3787 (Z.L.); +86-136-8216-1911 (Y.L.)

Table S1 Analysis of variance (ANOVA) of toluene adsorption capacity (Y_1).

Soruce of Variation	Quadratic Sum	Defree of Freedom	Mean Square	F	P	significance
Model	47,945.57	9	5327.29	209	<0.0001	Significant
A	18,956.64	1	18,956.64	743.7	<0.0001	-
B	74	1	74	2.9	0.1192	-
C	2491.65	1	2491.65	97.75	<0.0001	-
AB	261.06	1	261.06	10.24	0.0095	-
AC	168.36	1	168.36	6.61	0.0279	-
BC	371.28	1	371.28	14.57	0.0034	-
A^2	24,299.75	1	24,299.75	953.32	<0.0001	-
B^2	221.69	1	221.69	8.7	0.0146	-
C^2	2648.46	1	2648.46	103.9	<0.0001	-
Overall error	254.90	10	25.49	-	-	-
Lack of fit	112.71	5	22.54	0.79	0.5975	Non-significant
Purely error	142.19	5	28.44	-	-	-
R^2 = 0.9847		Adj R^2 = 0.9696		C.V. = 1.84%		

Table S2. Analysis of variance (ANOVA) of ethyl acetate adsorption capacity (Y_2).

Soruce of Variation	Quadratic Sum	Defree of Freedom	Mean Square	F	P	significance
Model	51,737.66	9	5748.629	162.8536	<0.0001	Significant
A	6716.229	1	6716.229	190.2648	<0.0001	-
B	463.9461	1	463.9461	13.14319	0.0046	-
C	2882.353	1	2882.353	81.65452	<0.0001	-
AB	549.4612	1	549.4612	15.56575	0.0028	-
AC	205.0313	1	205.0313	5.808355	0.0367	-
BC	15.40125	1	15.40125	0.436304	0.5238	-
A^2	29,345.94	1	29,345.94	831.3448	<0.0001	-
B^2	3375.008	1	3375.008	95.61101	<0.0001	-
C^2	14,183.93	1	14,183.93	401.8182	<0.0001	-
Overall error	352.99	10	35.30	-	-	-
Lack of fit	255.98	5	51.20	2.64	0.1553	Non-significant
Purely error	97.01	5	19.40	-	-	-
R^2 = 0.9832		Adj R^2 = 0.9667		C.V. = 3.43%		

Table S3. Analysis of variance (ANOVA) of yield (Y_3).

Soruce of Variation	Quadratic Sum	Defree of Freedom	Mean Square	F	P	significance
Model	107.03	9	11.89	15.88	<0.0001	Significant
A	85.58	1	85.58	114.23	<0.0001	-
B	1.09	1	1.09	1.45	0.2564	-
C	0.7	1	0.7	0.94	0.356	-
AB	0.5	1	0.5	0.67	0.433	-
AC	1.62	1	1.62	2.16	0.1722	-
BC	0	1	0	0	1	-
A^2	11.11	1	11.11	14.83	0.0032	-
B^2	0.18	1	0.18	0.24	0.6343	-
C^2	7.09	1	7.09	9.46	0.0117	-
Overall error	7.49	10	0.75	-	-	-
Lack of fit	4.20	5	0.84	1.27	0.3983	Non-significant
Purely error	3.29	5	0.66	-	-	-
R^2 = 0.9346		Adj R^2 = 0.8757		C.V. = 2.31%		

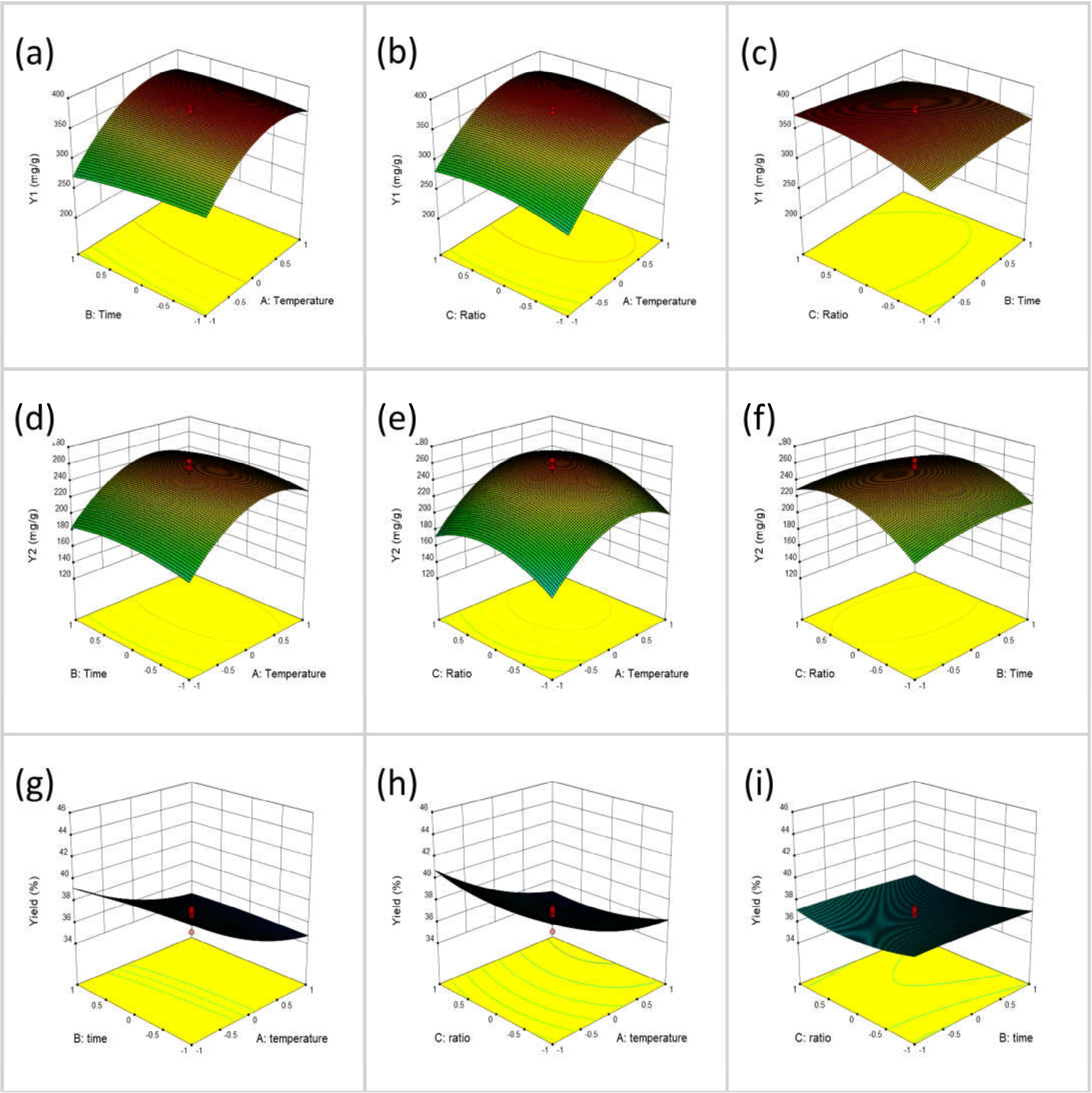

Figure S1. 3D surface of surface response plots for toluene adsorption capacity (**a**–**c**), ethyl acetate adsorption capacity (**d**–**f**), and carbon yield (**g**–**i**).

The briefly introduction of Langmuir equation, Freundlich equation, Sips equation, Toth equation, and Redlich-Peterson equation.

The Langmuir isotherm

Langmuir equation assumes monolayer adsorption which takes place at homogeneous interface. This equation describes the adsorption isotherms well in low and medium pressure range. It can be expressed as Equation (1).

$$q_e = \frac{q_m K_A C_e}{1 + K_A C_e} \quad (1)$$

where,q_e(mg/g) is the equilibrium adsorption capacity,C_e(mg/m^3) is the equilibrium concentration of adsorbate, q_m(mg/g) is the maximum saturation adsorption capacity, and K_A is Langmuir constant. $K_A$depends on the temperature and the properties of both adsorbent and adsorbate. A large K_A indicates stronger adsorption capacity of the adsorbent.

The Freundlich isotherm

Freundlich equation is widely used for multi-layer adsorption. It assumes that the adsorption takes place on non-uniform interface. Freundlich equation is suitable for adsorption at low concentration. It can be expressed as Equation (2):

$$q_e = k_f C_e^n \tag{2}$$

where, k_f is the Freundlich constant related to adsorption capacity and adsorption strength;n is the factor of heterogeneity. The smaller the n, the better the adsorption performance of the adsorbents. The adsorption will be easy when nis in the range of 0.1 to 0.5, while the adsorption will become difficult when n is greater than 2.0.

The Sips isotherm

Sips is a three-parameter equation combining Langmuir equation and Freundlich equation. It can be used to calculate the adsorption capacity of heterogeneous surface adsorbents. Sips can be expressed as Equation (3):

$$q_e = \frac{a_m K_S C_e^n}{1 + K_S C_e^n} \tag{3}$$

where, a_m,K_S, and n are the Sips constants. n represents the heterogeneity of the adsorbent surface.

The Toth isotherm

Toth isotherm is an improved form of Langmuir isotherm. The inhomogeneous energy parameter, d, is introduced so that this equation can be applied in wide pH range. It can be expressed as Equation (4):

$$q_e = \frac{f C_e}{[g + (C_e)^d]^{1/d}} \tag{4}$$

where, f, g, and $d$are the Toth constants.

The Redlich-Peterson isotherm

Redlich-Peterson can be expressed as Equation a (5):

$$q_e = \frac{K_R C_e}{1 + \alpha_R C_e^{\beta}} \tag{5}$$

where, K_R, α_R, and β are the Redlich-Peterson empirically constants.
